# Examining academic performance across gender differently: Measurement invariance and latent mean differences using bias-corrected bootstrap confidence intervals

**DOI:** 10.3389/fpsyg.2022.896638

**Published:** 2022-08-05

**Authors:** Ioannis Tsaousis, Mohammed H. Alghamdi

**Affiliations:** ^1^Department of Psychology, National and Kapodistrian University of Athens, Athens, Greece; ^2^Department of Self Skills Development, King Saud University, Riyadh, Saudi Arabia; ^3^Education and Training Evaluation Commission (ETEC), Riyadh, Saudi Arabia

**Keywords:** general academic ability, academic performance, gender differences, measurement invariance, BC bootstrap confidence intervals

## Abstract

The aim of this study was threefold: First, to examine the dimensionality of the construct of General Academic Ability (GAA) at the subscale level providing additional insights over and above on the conceptualization of the construct. Second, to explore different degrees of measurement invariance of the GAA across gender using more recent advancements in the examination of Measurement Invariance (i.e., Bias-Corrected bootstrap Confidence Intervals). Third, to examine gender differences across the different facets of the GAA at the latent mean level. The sample consisted of 1,800 high school graduates who applied for higher education in Saudi Arabia. The results from the analysis indicated that the hierarchical model with one higher-order factor (i.e., general academic ability) and four lower-order cognitive factors (i.e., verbal ability, quantitative ability, scholastic aptitude, and GPA) exhibited an excellent fit to the data. In terms of the measurement invariance hypothesis, it was found that the hierarchical model exhibits full configural and metric invariance and partial scalar invariance. Finally, using the Latent Mean Difference procedure, the results showed gender differences in the Verbal and GPA domains. Although significant differences were also found in the Scholastic aptitude domain, this finding is not stable due to several non-invariant items within the domain. In both cases, females scored higher than males. Finally, regarding the higher-order factor (GAA), the results showed that females scored higher than males. There were no significant differences in the Quantitative domain.

## Introduction

Academic performance is a crucial research topic, particularly for college admission purposes. Though there is no agreed-upon of its measures, literature determined three group factors that affect the quality of academic performance: Aptitude, instruction, and environment (Walberg, 1981). For admission purposes, the most common way of measuring academic performance is through standardized tests. Literature suggests that these tests are affected by several individual and contextual factors. For example, researchers found that ethnicity is one of the factors that affect academic performance. White students are more likely to have a higher cumulative general point average (CGPA; Weissberg et al., 2003; Laskey and Hetzel, 2011; Bronkema and Bowman, 2019) than students of color or other ethnicities. Parental status is another factor that affects academic success. Several studies have demonstrated that parental education significantly predicts cumulative general point average (CGPA; Kelly, 2004; Dayioğlu and Türüt-Aşik, 2007; Dika, 2012). Another factor that is affecting academic performance is age. Several researchers found that age significantly predicts both first semester and first-year GPA (Gibbison et al., 2011; Ferrão and Almeida, 2019). Finally, pre-college academic preparation was found to be influential for academic success. Several studies affirmed that students with higher high school grade point average (HSGPA) were more likely to achieve a higher college grade point average (CGPA; Dayioğlu and Türüt-Aşik, 2007; Dika, 2012; Komarraju et al., 2013; Wurf and Croft-Piggin, 2015; Saunders-Scott et al., 2018; Sulphey et al., 2018; Bronkema and Bowman, 2019; Caviglia-Harris and Maier, 2020).

Gender is another factor assumed to affect students’ academic scores considerably. Many studies have shown that boys and girls perform differently (e.g., O’Reilly and McNamara, 2007; Abubakar and Bada, 2012). However, contrary to the compelling evidence regarding the effect of other individual and contextual variables on academic performance, the gender role in academic performance is still an issue that attracts increased scientific interest, mainly due to the inconclusive reported findings. For example, studies pointed out no significant gender difference in students’ academic achievement. In a meta-analytic study, Else-Quest et al. (2010) showed negligible gender differences in the results of standardized mathematics tests. Similarly, Ajai and Imoko (2015) found that male and female students did not significantly differ in achievement and retention scores in mathematics.

On the other hand, other studies have reported significant differences, with the boys or the girls performing better. For example, several studies suggest that females outperform males in language-based subjects and verbal tests (e.g., Deary et al., 2007; Spinath et al., 2010), and males outperform their female counterparts in STEM-related subjects (e.g., math, engineering, etc.) and visuospatial tests (e.g., Strand et al., 2006; Lakin, 2013). Evidence from TIMSS 2015 indicated that 4th-grade boys outperformed girls in mathematics in about one-third of the countries that took place, while for 8th grade, this figure is reduced to one-sixth (Mullis et al., 2016). In a meta-analytic study, Voyer and Voyer (2014) found that females appear to have higher school grades in language-based subjects and STEM subjects than males.

Gibb et al. (2008) introduced a biological perspective and the way the human brain is organized across gender as a possible explanation for obtained gender differences in achievement, while Carvalho (2016) highlighted the role of personality dimensions (e.g., aggressiveness) as potential mediators in the relationship between gender and academic performance.

The gender gap in academic performance has been examined not only in terms of school grades and performance but also in terms of other aspects of academic life. For example, Bugler et al. (2015) suggested that females have higher motivation levels and better adaptation. Similarly, Ghazvini and Khajehpour (2011) found that female students showed more internal locus of control in academic performance than male students. However, no differences were found in academic self-concept as a function of gender. They also found that boys use learning strategies to a lesser degree than do girls and that girls take greater responsibility for their academic failures.

A possible explanation for the inconsistencies in the obtained results might lie in the method of analysis applied by the researchers to examine the phenomenon. For example, almost all reported studies have used conventional mean difference statistics (e.g., *t*-test, ANOVA, etc.) to examine possible gender differences in academic achievement. However, this approach is based on the assumption that the measurement properties of the scale used to measure academic performance are equivalent across the compared groups (Vandenberg and Lance, 2000). Despite its appeal, this assumption is highly problematic since it is not always true. Thus, findings that are based on this assumption could be questioned since one can never be sure that the reported differences are due to actual group differences or are due to the different conceptualization (i.e., factor structure) of the construct being measured across groups (Reise et al., 1993).

An alternative and more robust statistical technique for comparing groups would be examining the latent means derived from the multi-group comparisons performed *via* structural equation modeling and measurement invariance techniques (Cheung and Rensvold, 2002; Millsap, 2011). Measurement invariance is determined if the trait scores between different groups are comparable and have the same meaning. On the other hand, although determining measurement invariance across groups is a logical prerequisite to conducting meaningful cross-group comparisons (e.g., males vs. females), measurement invariance is rarely tested in psychological research (Horn and McArdle, 1992).

Based on the above and given the lack of agreement among researchers on the role of gender in academic performance, the aims of this study were threefold: first, to provide additional insights over and above on the conceptualization of the construct of General Academic Ability (GAA) as introduced and discussed by Dimitrov et al. (2015). Second, to explore different degrees of measurement invariance of the GAA construct across gender. Using more recent advancements in Measurement Invariance (i.e., Bias-Corrected bootstrap Confidence Intervals), we will attempt to understand the factorial structure of the GAA in more depth. Third, to examine gender differences across the different facets of the GAA at the latent mean level.

## Materials and methods

### Participants and procedure

The sample consisted of 1,800 high school graduates who applied for higher education in Saudi Arabia. Along with other admission requirements that Saudi higher education institutions have toward their applicants, a key factor in the decision on acceptance is a composite score of the applicants based on their high school grade (GPA) and test scores on two standardized tests, namely General Aptitude Test (GAT) and Standardized Achievement Admission Test (SAAT). Nine hundred (50.0%) of the participants were males, and 900 (50.0%) were females, with a mean age of 17.50 years (SD = 1.12), ranging between 17 and 32 years. Regarding the type of school, 1,259 individuals (69.1%) graduated from a public school and 541 (30.1%) from a private school. In terms of their socio-economic status, 548 (30.4%) participants were coming from families with <5,000$ annual income, 554 (30.8%) from families with 5,000–1,000$ annual income, 321 (17.8%) from families with 10,000–15,000$ annual income, 211 (11.7%) from families with 15,000–20,000$ annual income and 166 (9.2%) from families with annual income >20,000$. In terms of origin, all participants were coming from the 13 regions of Saudi Arabia. Although not representative, the characteristics of a sample approximate the characteristics of the Saudi population. Ethical approval for the present study was granted by the Education and Training Evaluation Commission’s Ethics Committee (Ref No.TR332-2021). All participants were informed that their responses would be utilized as a part of a larger study to evaluate the psychometric properties of the measure. Completion of the test comprised their informed consent for their participation. No participants reported any psychological or emotional issues that would inhibit their full performance.

### Measures

#### The general aptitude test for science major

This is a general cognitive ability test developed developed by the Qiyas (Education & Training Evaluation Commission), which measures two different cognitive areas: (a) verbal and (b) numerical. This GAT version contains 95 dichotomous items (right-wrong) and measures seven different cognitive skills: (i) word meaning (12 items), (ii) sentence completion (15 items), (iii) analogy (15 items), (iv) reading comprehension (23 items), (v) arithmetic (17 items), (vi) analysis (seven items), and (vii) geometry (six items). The first four subscales measure verbal ability and the remaining numerical ability. A global cognitive ability factor composed of the scores from the two domain scales (i.e., verbal and numerical) is also available. There are five different versions of the GAT (Form A to E). Previous studies have shown that GAT is a very reliable test, with alpha reliabilities ranging from 0.73 to 0.82 for both sections (Verbal and Numerical; e.g., Dimitrov, 2013; Dimitrov et al., 2015; Dimitrov and Shamrani, 2015). Additionally, several studies have shown that GAT is an instrument with solid psychometric properties (e.g., Alanazi, 2014; Alamoudi et al., 2021; Aldurayheem, 2022).

#### The standard achievement admission test

Standard achievement admission test is an admission test developed by the Qiyas (Education & Training Evaluation Commission), covering four basic subject areas: Biology, Chemistry, Physics, and Mathematics, and focuses on the material of the official 3-year (scientific) curriculum of the Saudi High Schools. Standard achievement admission test is comprised of 88 multiple-choice items (four alternative options), which are distributed as follows: 20% of each subject for the first year of the high school syllabus, 30% of each subject for the second year of the high school syllabus, and 50% of each subject for the third year of the high school syllabus. The test time for each section is 25 min. Previous studies reported reliability indices ranging from 0.62 to 0.74 (e.g., Tsaousis et al., 2018). Additionally, several studies have demonstrated that SAAT is a valid predictor for various outcome criteria (e.g., Alamoudi et al., 2021; Alsagoafi, 2021; Vista and Alkhadim, 2022).

#### High school grade point average

The Ministry of Education provided the high school grades for the 1st and 2nd semesters and the Grade Point Average for each student at the end of the academic year.

### Data analysis strategy

To test the latent structure of the GAA, a hierarchical (second-order) factor model with 16 observed variables that are a function of four lower-order cognitive factors (i.e., verbal ability, quantitative ability, school scholastic aptitude, and GPA) and one higher-order factor (GAA) that accounts for the commonality shared by the lower order factors, was examined. We used the following indices to assess model fit to the data: the chi-square (*χ*^2^) statistic and the related degrees of freedom (df), the Comparative Fit Index (CFI), the Tucker–Lewis Index (TLI), the Root Mean Square Error of Approximation (RMSEA), and the Standardized Root Mean Square Residual (SRMR). A CFI and TLI value above 0.90 indicates an acceptable fit (with values >0.95 being ideal; Brown, 2015). Further, RMSEA and SRMR values up to 0.08 indicate a reasonable fit to the data, while values up to 0.05 indicate an excellent fit (Hu and Bentler, 1999). Maximum likelihood with robustness to non-normality and non-independence of observations (MLR; Muthén and Muthén, 1998-2016) was the model’s estimation method.

To test whether GAA sub-scales exhibited measurement invariance across gender, a multi-group confirmatory factor analysis (MGCFA) was conducted (Vandenberg and Lance, 2000; Milfont and Fischer, 2015). Τhree different (nested) models were examined: configural, metric, and scalar invariance. In configural invariance, the extent to which the scale’s factor structure is conceptualized similarly across groups was tested. To do that, the item factor loadings and item thresholds were allowed to vary across both groups (unconstrained model). This model also serves as the baseline model with which the other two models are compared for invariance. Next, apart from fixing construct dimensionality to be invariant, additional equality constraints were imposed on the item factor loadings across the two groups. In that way, the metric (or weak) invariance was tested. If a scale exhibits metric invariance, the respondents across groups attribute the same meaning to the latent construct under study. Then, additional constraints to factor loadings and item intercepts were imposed to test for scalar invariance. Establishing scalar invariance indicates that participants with the same score on the latent construct would obtain the same score on the observed variable irrespective of their group membership (Putnick and Bornstein, 2016). When scalar invariance is established, comparisons among group means are meaningful (Vandenberg and Lance, 2000; Millsap, 2011).

Finally, latent mean differences were evaluated to examine for possible gender differences. In an SEM framework, to estimate the difference between two group means at a latent level, one of the groups should be served as a reference group, and its mean should be fixed to zero. In this case, the latent mean of the other group represents the difference between the latent means of the two groups (Hong et al., 2003). Thus, a significant mean of a compared group would indicate that this group has a different level of the latent construct relative to the reference group. In this analysis, females were chosen as reference groups (coded as 0).

It should be noted that Cheung and Rensvold (2002) suggest a protocol of eight steps when the measurement invariance of a scale is examined: apart from the configural, metric, and scalar invariance, and before the examination of latent means, additional constraints could be imposed to examine invariance of uniqueness variances, factor variances, factor covariances or path coefficients across groups. However, these additional forms of invariance are useful only when specific hypotheses regarding the relationship among the dimensions of the measured construct may be of interest (Cheung and Rensvold, 2000; Byrne, 2011). In this study, these additional steps were omitted since the main objective of this study was the examination of gender differences at the latent mean level. According to Cheung and Lau (2012), only scalar invariance is a prerequisite for comparing latent means.

The most frequently used method for evaluating invariance among different consecutive models is examining the changes in various fit indices such as ΔCFI, ΔRMSEA, and ΔSRMR (e.g., Vandenberg and Lance, 2000; Chen, 2007; Cheung, 2008). However, this method raises several issues which put in question its effectiveness in testing invariant parameters. For example, almost all fit indices are based on the *χ*^2^ statistic; it is widely known, however, that this statistic is sensitive to sample size (Kelloway, 1995). Thus, in large sample sizes, even small differences in any parameter across groups may lead to the rejection of the null hypothesis of invariance. Another major limitation is that criteria such as ΔCFI have no known sampling distribution, and therefore, it is not possible to establish any significance testing for evaluating invariant parameters. As a result, several scholars argue that the suggested cut-off criteria seem arbitrarily defined. Some other scholars argue that it is not statistically correct to apply the same cut-off criteria for evaluating models with different levels of complexity (e.g., Cheung and Rensvold, 2002) since more complex models may need different cut-off values compared to less complex models.

Another concern is related to the sample size of the compared groups. It has been argued that several fit indices (e.g., CFI, RMSEA, SRMR) and their corresponding changes in fit across the different invariance models (e.g., ΔCFI, ΔRMSEA, ΔSRMR) are less powerful when the sample sizes are equal rather than equal across groups (Chen, 2007). This may be because the sample with the larger sample size affects more the parameter estimation process of the constrained model.

An issue of equal importance is when full measurement invariance cannot be established, and as a result, latent means cannot be examined. One solution to this problem is to relax one by one the invariance constraints based on the modification indices (MIs), starting with the parameter with the largest MI until the minimum cut-off criterion is met. In this case, a partial metric or scalar invariance is established, and this model can then be used to test latent means (Marsh and Hocevar, 1985; Byrne et al., 1989). However, Cheung and Rensvold (1998) argue that all constrained parameters in a fully constrained model must be invariant apart from the relaxed one to get robust MI values. If more than one of the constrained parameters is non-invariant, there is an increased risk for inaccurate identification of non-invariant items.

An alternative approach to examining measurement invariance that addresses most of the abovementioned issues is using the Bias Corrected (BC) bootstrap confidence intervals (Cheung and Lau, 2012). This approach automatically generates 1,000 bootstrap samples (or more), and parameter differences (e.g., in factor loadings or intercepts) between groups are calculated across samples. Then, confidence intervals for each bootstrap sample are calculated, adjusting the bootstrap distribution of the parameters. This idea is based on past work where BC bootstrap confidence intervals have been used to test the significance of factor loading differences (Meade and Bauer, 2007) and differences in indirect effects across groups (Lau and Cheung, 2012).

This method exhibits several advantages over the standard approach of testing measurement invariance. The most important one is that it provides more accurate parameter estimates since it does not calculate the estimates from the original data but rather is based on bootstrap resamples. Moreover, the so-called bias-corrected approach corrects for bias and skewness in the distribution of bootstrap estimates. Another important advantage is that it provides a significance test for evaluating the magnitude of the difference in parameters across groups, avoiding arbitrary conclusions from unjustified cut-off criteria. Moreover, it allows examining multiple parameters across groups simultaneously as it provides a separate table with all estimated parameters. Finally, the BC bootstrap confidence intervals method is less restricted in the normality assumption than standard methods used in measurement invariance. In addition, it overcomes issues related to sample size across groups since it is based on a resampling technique (Cheung and Lau, 2012). All analyses were conducted with Mplus version 8.2 (Muthén and Muthén, 1998-2016).

## Results

Descriptive statistics, normality indices, and inter-correlations among the study variables are presented in Table 1. As can be seen, all variables had skewness and kurtosis values within the acceptable range (i.e., <2.0), indicating that the data are normally distributed. Additionally, all variables are intercorrelated to a considerable degree, especially the variables within each domain (i.e., GAT Verbal, GAT Quantitative, SAAT, and GPA).

**Table 1:** Descriptive statistics and inter-correlations among the variables of the study (*N* = 1,800).

Subscales	1	2	3	4	5	6	7	8	9	10	11	12	13	14	15	16
1. Q_AR																
2. Q_GE	0.60															
3. Q_AN	0.56	0.55														
4. Q_AL	0.35	0.31	0.29													
5. Q_CO	0.43	0.43	0.37	0.22												
6. V_AN	0.56	0.52	0.49	0.30	0.40											
7. V_CA	0.53	0.48	0.42	0.27	0.37	0.59										
8. V_SC	0.45	0.40	0.36	0.20	0.32	0.50	0.48									
9. V_RC	0.59	0.52	0.46	0.30	0.42	0.62	0.64	0.51								
10. BIO	0.63	0.57	0.54	0.35	0.45	0.58	0.51	0.46	0.60							
11. CHEM	0.58	0.51	0.48	0.28	0.40	0.55	0.51	0.47	0.59	0.72						
12. PHYS	0.59	0.53	0.49	0.31	0.44	0.55	0.54	0.46	0.63	0.72	0.70					
13. MATH	0.60	0.52	0.47	0.30	0.42	0.61	0.59	0.52	0.66	0.71	0.73	0.72				
14. GPA1	0.44	0.37	0.35	0.22	0.29	0.37	0.38	0.30	0.40	0.43	0.41	0.43	0.42			
15. GPA2	0.39	0.33	0.30	0.21	0.26	0.33	0.32	0.26	0.35	0.38	0.36	0.38	0.37	0.88		
16. GPA3	0.51	0.44	0.42	0.25	0.35	0.45	0.43	0.35	0.47	0.53	0.50	0.51	0.51	0.91	0.86	
Mean	9.22	5.08	4.44	2.11	4.07	8.43	5.53	3.16	11.05	13.61	10.33	10.49	12.11	90.91	92.95	89.82
SD	3.53	2.04	1.89	1.14	1.67	2.93	2.31	1.52	3.87	4.76	4.07	3.98	5.03	8.08	7.22	7.39
Skewness	−0.26	−0.36	−0.07	−0.07	0.03	0.02	0.05	−0.00	0.05	0.03	0.06	0.18	0.35	−1.57	−2.53	−0.86
Kurtosis	−0.81	−0.72	−0.64	−0.75	−0.48	−0.57	−0.74	−0.69	−0.69	−0.77	−0.65	−0.62	−0.64	7.10	17.69	0.77

Q_AR, Arithmetic; Q_GE, Geometry; Q_AN, Analysis; Q_AL, Algebra; Q_CO, Comparison; V_AN, Analogy; V_CA, Contextual Errors; V_SC, Sentence Completion; V_RC, Reading Comprehension; BIO, Biology; CHEM, Chemistry; PHYS, Physics; MATH, Mathematics; GPA, Grade Point Average; Q, GAT Quantitative Section; V, GAT Verbal Section. All correlation coefficients were significant at *p* < 0.001 level.

### GAA dimensionality results

**Figure 1:**
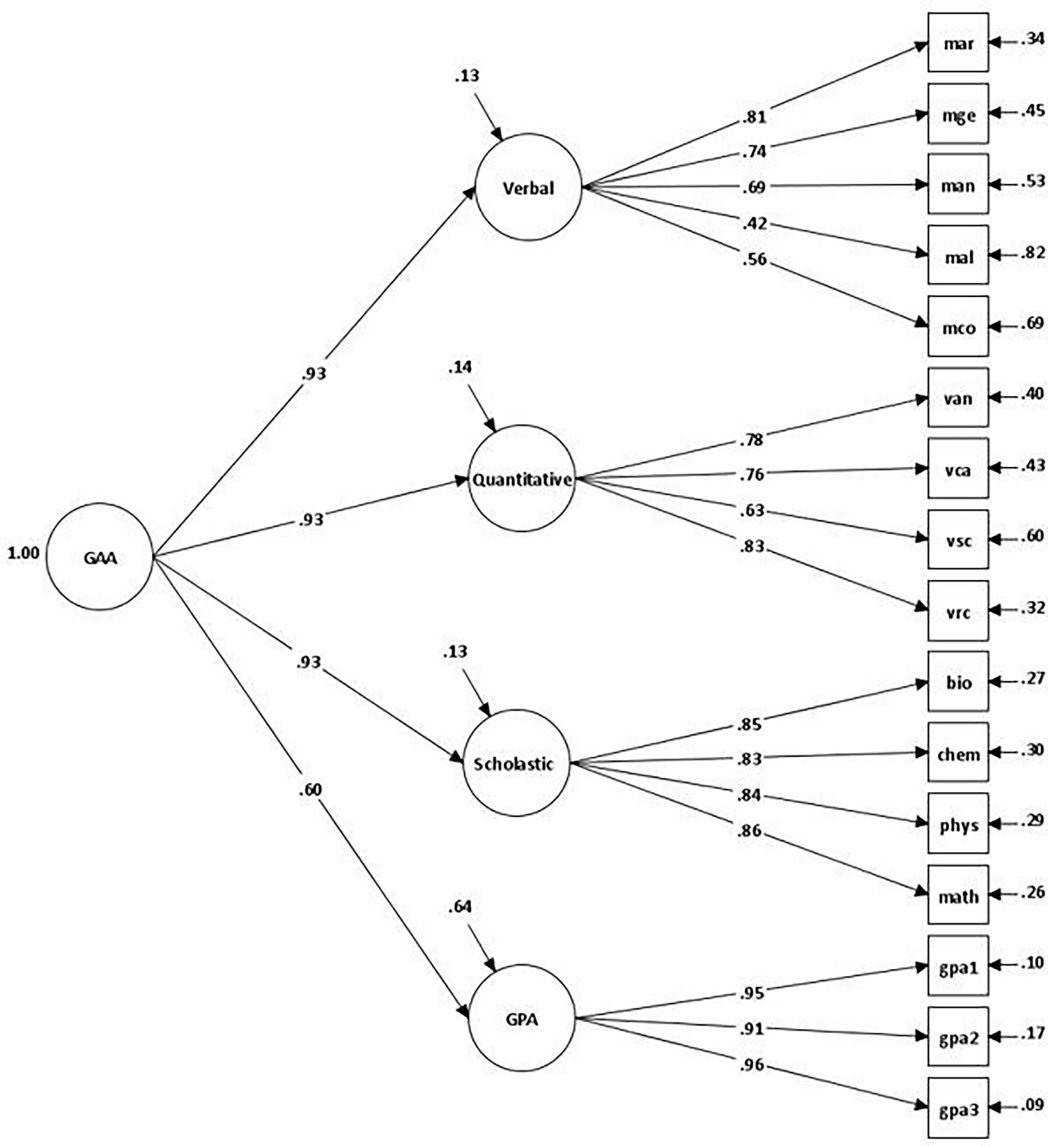
The hierarchical General Academic Ability (GAA) model.

Previous empirical evidence suggested a dominant GAA dimension underlies the students’ domain scores on GPA, GAT-Verbal domain, GAT-Quantitative domain, and SAAT total score (Dimitrov et al., 2015). In this study, we are expanding the investigation of the dimensionality of the GAA by using different subsets of indicators from the four cognitive domains: five indicators for the Quantitative domain (i.e., arithmetic, geometry, analysis, algebra, and comparison), four indicators for Verbal domain (i.e., analogy, contextual errors, sentence completion, and reading comprehension), four scholastic aptitude indicators (i.e., biology, chemistry, physics, and mathematics), and three indicators of school performance across the academic year (1st semester, 2nd semester, final grade). The results from the analysis indicated that the hierarchical model with one higher-order factor (i.e., general academic ability) and four lower-order cognitive factors (i.e., verbal ability, quantitative ability, scholastic aptitude, and GPA) exhibited an excellent fit to the data: *χ*^2^ = 546.68, df = 100; CFI = 0.971; TLI = 0.965; RMSEA (90% CIs) = 0.050 (0.046–0.054), and SRMR = 0.027. A graphical representation of this model is presented in Figure 1.

### Measurement invariance

First, configural invariance across groups was examined to test whether the overall factor structure (i.e., hierarchical model) holds for both groups. Results indicated that the hierarchical model fit the two groups well (Males: *χ*^2^ = 271.25, df = 100; CFI = 0.983; TLI = 0.979; RMSEA (90% CIs) = 0.044 (0.037–0.050), and SRMR = 0.027, and Females: *χ*^2^ = 359.57, df = 100; CFI = 0.960; TLI = 0.952; RMSEA (90% CIs) = 0.054 (0.048–0.060), and SRMR = 0.031). Next, the Bias-Corrected (BC) bootstrap confidence intervals technique was applied to test metric invariance across groups. For identification purposes, one item from each latent variable was selected (i.e., the first indicator) as a referent indicator, and the factor loading of this indicator was fixed to 1. The results from the analysis indicated an excellent model fit to the data: *χ*^2^ = 612.49, df = 200; CFI = 0.980; TLI = 0.976; RMSEA (90% CIs) = 0.048 (0.044–0.052), and SRMR = 0.029. To examine whether all factor loadings across gender groups were equal, new parameters were defined as the difference in the factor loadings across groups, and a BC bootstrap confidence interval for this difference was generated (Table 2). It should be noted that 99% BC bootstrap confidence intervals were chosen to test the significance of the new parameters. When zero is located within the confidence interval, the null hypothesis of invariance is accepted. As can be seen, all new parameters related to the four latent variables are not significantly different across male and female groups. Thus, metric invariance exists for this construct.

**Table 2:** Bias-Corrected bootstrap confidence intervals for testing metric measurement invariance (*N* = 1,800).

New estimated parameter	Pair contrasted	99% CI lower bound	Estimate	99% CI upper bound
LD1	Q_GE F vs. Q_GE M	−0.095	−0.021	0.045
LD2	Q_AN F vs. Q_AN M	−0.062	0.021	0.095
LD3	Q_AL F vs. Q_AL M	−0.030	0.023	0.069
LD4	Q_CO F vs. Q_CO M	−0.062	0.010	0.074
LD5	V_CA F vs. V_CA M	−0.038	0.067	0.177
LD6	V_SC F vs. V_SC M	−0.029	0.049	0.123
LD7	V_RC F vs. V_RC M	−0.231	−0.049	0.145
LD8	CHEM F vs. CHEM M	−0.074	0.019	0.116
LD9	PHYS F vs. PHYS M	−0.117	−0.021	0.073
LD10	MATH F vs. MATH M	−0.085	0.039	0.163
LD11	GPA2 F vs. GPA2 M	−0.017	0.077	0.205
LD12	GPA3 F vs. GPA3 M	−0.218	−0.062	0.079
LD13	F2 F vs. F2 M	−0.134	−0.005	0.121
LD14	F3 F vs. F3 M	−0.198	0.035	0.214
LD15	F4 F vs. F4 M	−0.194	0.134	0.492

LD, Difference in loadings across groups; Q_GE, Geometry; Q_AN, Analysis; Q_AL, Algebra; Q_CO, Comparison; V_CA, Contextual Errors; V_SC, Sentence Completion; V_RC, Reading Comprehension; CHEM, Chemistry; PHYS, Physics; MATH, Mathematics; GPA2, Grade Point Average for 2nd semester; GPA3, Grade Point Average final; Q, GAT Quantitative Section; V, GAT Verbal Section; F, Females; M, Males.

Next, scalar invariance across groups was examined by constraining the item intercepts to be invariant across the contrasted groups. The results from the analysis indicated an excellent model fit to the data: *χ*^2^ = 659.43, df = 215; CFI = 0.978; TLI = 0.976; RMSEA (90% CIs) = 0.048 (0.044–0.052), and SRMR = 0.061. Again, new parameters were defined as the difference in the item intercepts across groups, and then a BC bootstrap confidence interval for this difference was estimated. The results from this analysis are presented in Table 3. As can be seen, although the scalar model had an excellent fit, a closer examination of the individual scalar parameters did not support full scalar invariance. Items Q_GE (Geometry) from the Quantitative domain, V_CA (Contextual Errors) from the Verbal domain, CHEM (Chemistry), and MATH (Mathematics) from the scholastic aptitude domain, and GPA3 (final GPA) were not invariant across groups.

**Table 3:** Bias-Corrected bootstrap confidence intervals for testing scalar measurement invariance (*N* = 1,800).

New estimated parameter	Pair contrasted	99% CI lower bound	Estimate	99% CI upper bound
ID1	Q_GE F vs. Q_GE M	0.061	0.267	0.501
ID2	Q_AN F vs. Q_AN M	−0.018	0.188	0.398
ID3	Q_AL F vs. Q_AL M	−0.059	0.069	0.203
ID4	Q_CO F vs. Q_CO M	−0.004	0.178	0.372
ID5	V_CA F vs. V_CA M	−0.569	−0.314	−0.052
ID6	V_SC F vs. V_SC M	−0.158	0.024	0.177
ID7	V_RC F vs. V_RC M	−0.557	−0.171	0.269
ID8	CHEM F vs. CHEM M	−0.291	0.094	0.516
ID9	PHYS F vs. PHYS M	−1.167	−0.785	−0.402
ID10	MATH F vs. MATH M	−1.638	−1.179	−0.693
ID11	GPA2 F vs. GPA2 M	−0.922	−0.407	0.049
ID12	GPA3 F vs. GPA3 M	−1.408	−0.839	−0.390

ID, Difference in intercepts across groups; Q_GE, Geometry; Q_AN, Analysis; Q_AL, Algebra; Q_CO, Comparison; V_CA, Contextual Errors; V_SC, Sentence Completion; V_RC, Reading Comprehension; CHEM, Chemistry; PHYS, Physics; MATH, Mathematics; GPA2, Grade Point Average for 2nd semester; GPA3, Grade Point Average final. Q, GAT Quantitative Section; V, GAT Verbal Section; F, Females; M, Males.

As discussed earlier, if the invariance of item intercepts is not supported, partial scalar invariance could be established as an alternative. An important consideration is how many items are allowed to be invariant to establish partial measurement invariance (metric or scalar). Several scholars suggest that robust partial invariance is accomplished if the majority of the items in a latent variable are invariant across groups (Reise et al., 1993; Vandenberg and Lance, 2000; Cheung and Lau, 2012). This assumption is met for all latent variables in our study, apart from the scholastic aptitude variables (two out of four variables were non-invariant). Thus, for this latent variable, the comparison of latent means across gender was not conducted. For all the other latent variables, latent means using the BC bootstrap confidence interval approach were performed.

Given that the configural, metric, and partial scalar invariance assumptions were satisfied, the next step was to test for possible gender differences at the latent mean level. Since partial scalar invariance was established in the previous step, the non-invariant items (i.e., Q_GE, V_CA, CHEM, MATH, and GPA3) were left to be freely estimated. This model demonstrated an acceptable model fit, indicating that partial scalar invariance was achieved (*χ*^2^ = 685.27, df = 215; CFI = 0.977; TLI = 0.974; RMSEA (90% CIs) = 0.049 (0.045–0.053), and SRMR = 0.055). The results from the analysis showed that there were gender differences in the Verbal domain and the GPA domain (Table 4). Although significant differences were also found in the Scholastic aptitude domain, as mentioned earlier, this finding is not stable due to a large number of non-invariant items within the domain. In both cases, females scored higher than males. There were no significant differences in the Quantitative domain. In terms of the higher-order factor (GAA), the results showed a significant gender effect and that females scored higher than males.

**Table 4:** Bias-corrected bootstrap confidence intervals for comparing GAA latent means across gender.

Domain	99% CI lower bound	Estimate	99% CI upper bound
Verbal ability	−1.091	−0.679	−0.306
Quantitative ability	−0.141	0.160	0.473
Scholastic aptitude	−2.684	−2.042	−1.527
Grade point average	−2.186	−1.216	−0.128
General academic ability	−0.522	−0.843	−1.272

## Discussion

The main aim of this study was threefold: first, to examine the factorial structure of the General Academic Ability (GAA), a construct developed to measure the academic performance of high school graduates in Saudi Arabia and used as the key criterion in the decision on acceptance to higher education in Saudi Arabia. The second aim of this project was to explore different degrees of measurement invariance of the GAA construct across gender. Using more recent advancements in Measurement Invariance (i.e., Bias-Corrected bootstrap Confidence Intervals), we will attempt to understand the factorial structure of the GAA in more depth. Finally, the third aim of this study was to examine whether there are gender differences across the different facets of the GAA at the latent mean level.

Dimitrov et al. (2015) introduced the GAA higher-order factor as a function of four different composites: (a) verbal ability, (b) quantitative ability, (c) scholastic aptitude, and (d) Grade Point Average (GPA). Previous findings provided sufficient evidence to support the psychometric quality of the construct and its usefulness as a key criterion for university entrance. However, this information originated from the analysis at the domain-only level (four main variables). Therefore, in this study, we attempted to provide additional insights on the conceptualization of the construct by examining its factor structure using as indicators the subscales from which these four domains were composed, that is 4 for verbal ability, 5 for quantitative ability, 4 for scholastic aptitude, and 3 for GPA (total of 16 variables).

To examine the factor structure of the GAA, a hierarchical model with one higher-order factor (i.e., general academic ability) and four lower-order cognitive factors (i.e., verbal ability, quantitative ability, scholastic aptitude, and GPA) was examined. The results from the analysis showed an excellent fit to the data, providing further support to the findings reported by Dimitrov et al. (2015). The GAA is a higher-order construct that underlies the students’ scores on several cognitive criteria coming from standardized tests and school performance. In this context, this study demonstrated that these criteria are valid indicators of this higher-order dimension and could be used as a valid criterion for entrance to higher education.

The second aim of this study was to examine whether GAA exhibits measurement invariance across gender. In other words, whether males and females conceptualize the latent constructs in identical ways (configural), respond to the items in the same way (metric), and have the same score on each latent construct obtain the same score on the observed variable regardless of their group membership (scalar). Previous research has demonstrated that standard methods typically used to test measurement invariance and identify non-invariant items (e.g., the likelihood ratio test – LRT across compared models or the change in goodness-of-fit indices among constrained models such as ΔCFI) share certain limitations and pitfalls (e.g., the sensitivity of the Δ*χ*^2^ to large sample sizes, arbitrarily determined cut-off values in the reduction of a fit index across compared models, lack of any significance test; see Cheung and Lau, 2012).

In this study, we used an alternative approach *via* the Bias-Corrected (BC) bootstrap confidence intervals. BC bootstrap confidence intervals have been previously used in other statistical procedures, including estimating factor loading differences (Meade and Bauer, 2007) or evaluating differences in indirect effects across groups (Lau and Cheung, 2012). In this study, we use this approach as an extension of the above applications. Some of the most important advantages of this approach in testing measurement invariance are that it allows us to estimate more accurate parameter estimates and the presence of a robust significance test to identify non-invariant items.

The results showed that the hierarchical model exhibits full configural and metric invariance and partial scalar invariance. However, items Q_GE (Geometry) from the Quantitative domain, V_CA (Contextual Errors) from the Verbal domain, CHEM (Chemistry) and MATH (Mathematics) from the scholastic aptitude domain, and GPA3 (final GPA) were not invariant across gender. This finding suggests that males and females with the same score on each latent construct do not obtain the same score on these observed variables (i.e., one group might find these subscales easier than the other group). This finding is particularly important since it contributes to the better justification of the validity of the factorial structure of the GAA. It should also be noted that when standard approaches for testing measurement invariance were applied (i.e., LRT and ΔCFI, ΔRMSEA), all observed variables were invariant across gender.

Next, differences in academic performance between female and male students were examined by estimating Latent Mean differences. Although only partial scalar invariance was achieved at the previous level of analysis, it is possible to conduct valid cross-group comparisons across gender between different latent domains since the majority of the items in a latent variable were invariant across groups (Reise et al., 1993; Vandenberg and Lance, 2000).

Gender differences or “gender gap” in academic achievement have continuously attracted scientific interest not only in educational research but also from a political and economic context (e.g., Hausmann et al., 2009; UNESCO, 2015) and have raised important questions with educational, political, and economic consequences (e.g., Klasen, 2002; EGREES, 2005). In previous years, international organizations and scientific collaborations have attempted to highlight the issue and provide convincing evidence regarding the role of gender in academic achievement (e.g., Nguyen and Ryan, 2008; UNESCO, 2015; OECD, 2016). Unfortunately, these attempts have not returned consistent results resulting in ambiguity and vagueness in terms of the factors that might cause this inconsistency. This paper adds to existing research into the gender gap in academic attainment by proposing the application of a novel statistical approach to re-examine a research question that has been attracting researchers’ interest continually and has not been clarified yet, mainly due to the inconclusive findings: The Bias-Corrected (BC) bootstrap confidence intervals. Some of the most important advantages of this approach in testing measurement invariance across groups are that it allows us to estimate more accurate parameter estimates and uses a robust significance test to identify non-invariant items. Therefore, we believe this statistical approach could offer more compelling evidence for the unresolved issue of whether there is a gender gap in academic attainment.

Using the Latent Mean Difference procedure, the results from this study showed gender differences in the Verbal domain and the GPA domain. In both cases, females scored higher than males. Although significant differences were also found in the Scholastic aptitude domain, this finding is not stable due to many non-invariant items within the domain. Regarding the higher-order factor (GAA), the results also showed that females scored higher than males. There were no significant differences in the Quantitative domain. The findings from this study support the general hypothesis that female students outperform their male counterparts in almost every aspect of academic life and provide further support to the gender gap in educational attainment. (e.g., Holmlund and Sund, 2008; Hausmann et al., 2009; Bugler et al., 2015). Most of the findings from this study are in line with previous results showing that females outperform males in language-based skills (e.g., Deary et al., 2007; Steinmayr and Spinath, 2008; Spinath et al., 2010) as well as that overall, they achieve higher average grades (Voyer and Voyer, 2014; Marcenaro-Gutierrez et al., 2017).

Although there are studies in the past arguing that males outperform females in STEM-related subjects such as mathematics and engineering (e.g., Strand et al., 2006; Lakin, 2013), more recent research using more advanced methodological and statistical techniques (e.g., meta-analysis) has demonstrated that are negligible or no gender differences in the results of standardized mathematics tests there is no gender effect in mathematical ability (Else-Quest et al., 2010; Ajai and Imoko, 2015). The findings from this study support the above argument.

The present study’s findings should be viewed in light of the below-mentioned limitations. One limitation is related to the characteristics of the participants, who were predominantly high school students and were Saudi in origin, which might constrain the generalizability of the findings. Further research is thus required to test the current findings in more diverse samples (e.g., university students, other cultures, etc.). Additionally, an issue not addressed in this study was the factorial invariance of the GAA across other groups. For example, it might be interesting to examine whether other demographic variables such as age could have long-run effects on academic performance. For example, Pellizzari and Billari (2012) found that older participants in a given class or a cohort perform better than younger participants. The same pattern of results has been reported by other researchers (e.g., Dhuey and Bedrad, 2006; Crawford et al., 2007). Thus, it would be interesting to compare possible GAA differences across high school students, university students, and more mature individuals (e.g., employees). It would also be interesting to examine the mechanisms leading to the gender gap in academic performance. Thus, future research might not only focus on detecting mean differences in predictor variables but also concentrate on the relations between potential predictors and specific individual (e.g., self-esteem, personality, etc.) as well as contextual (e.g., public vs. private schools, parenting, etc.) factors vary as a function of gender. For example, it would be interesting to examine parental factors such as parenting style (Masud et al., 2019) or parental involvement and support (Topor et al., 2010; Jeynes, 2016) as possible determinants of the observed gender inequality in academic performance.

Overall, measurement invariance is an important research topic when researchers hope to investigate whether the measurement model of the latent constructs (including pattern and magnitude) holds across groups and whether the mean levels of the latent constructs hold across groups. The results from this study showed that there are gender differences in academic ability and that females generally outperform males in verbal abilities and GPA. However, no gender differences were found in terms of quantitative skills.

## Data availability statement

The raw data supporting the conclusions of this article will be made available by the authors, without undue reservation.

## Ethics statement

The studies involving human participants were reviewed and approved by Education and Training Evaluation Commission’s Ethics Committee. The patients/participants provided their written informed consent to participate in this study.

## Author contributions

IT designed the research project, performed the statistical analysis, and completed the original version of the manuscript. MA contributed to the development of the article, polished, revised, and approved the final version of the manuscript. All authors contributed to the article and approved the submitted version.

## Conflict of interest

The authors declare that the research was conducted in the absence of any commercial or financial relationships that could be construed as a potential conflict of interest.

## Publisher’s note

All claims expressed in this article are solely those of the authors and do not necessarily represent those of their affiliated organizations, or those of the publisher, the editors and the reviewers. Any product that may be evaluated in this article, or claim that may be made by its manufacturer, is not guaranteed or endorsed by the publisher.
